# Small-Molecule Strategies for Polymyalgia Rheumatica and Giant Cell Arteritis in Older Adults

**DOI:** 10.3390/molecules31132218

**Published:** 2026-06-24

**Authors:** Jan Kurdybacha, Oleksii Kravets, Natalia Lekston, Kacper Kotyla, Olga Gumkowska-Sroka, Przemysław Kotyla

**Affiliations:** 1 Students’ Scientific Society, Department of Rheumatology and Clinical Immunology, Faculty of Medical Sciences in Katowice, Medical University of Silesia, 40-055 Katowice, Poland; 2 Department of Medical Biophysics, Medical Faculty in Katowice, Medical University of Silesia, 40-055 Katowice, Poland; 3 Department of Internal Medicine, Rheumatology and Clinical Immunology, Medical Faculty in Katowice, Medical University of Silesia, 40-055 Katowice, Poland

**Keywords:** polymyalgia rheumatica, giant cell arteritis, immunosenescence, Janus kinase inhibitors, small-molecule drugs, glucocorticoid-sparing

## Abstract

Polymyalgia rheumatica (PMR) and giant cell arteritis (GCA) are systemic inflammatory diseases deeply rooted in age-related immunosenescence and inflammaging. Conventional long-term glucocorticoid (GC) therapy poses significant metabolic and infectious risks for older adults, necessitating safer alternatives. This review critically evaluates the pathophysiological rationale and clinical efficacy of small-molecule drugs, including Janus kinase inhibitors (JAKi) and conventional synthetic disease-modifying antirheumatic drugs (csDMARDs), as steroid-sparing treatments for PMR and GCA. By selectively inhibiting intracellular networks like the JAK-STAT pathway and nucleotide biosynthesis, these agents aim to attenuate maladaptive inflammation. Clinical evidence highlights that JAK inhibitors, particularly upadacitinib for GCA and tofacitinib or baricitinib for PMR, demonstrate the potential to induce remission and significantly reduce the required GC burden in a subset of patients. Although methotrexate remains the primary csDMARD, its modest overall efficacy suggests it should be reserved for patients with definitive contraindications or restricted access to JAK inhibitors. Furthermore, novel therapies like clofutriben demonstrate potential in reversing GC-induced morbidities without compromising disease control. Ultimately, integrating targeted small-molecule immunomodulators establishes a crucial therapeutic paradigm that attempts to maximize clinical remission while safeguarding the physiological integrity of geriatric patients against severe GC toxicities.

## 1. Immunosenescence and Inflammaging: The Pathogenetic Foundation of Polymyalgia Rheumatica and Giant Cell Arteritis

Polymyalgia rheumatica (PMR) and giant cell arteritis (GCA) represent a spectrum of systemic inflammatory diseases with a strong predilection for advanced age. Aging of the immune system, commonly referred to as immunosenescence, plays a significant role in the pathomechanism of these conditions and provides a useful framework for understanding age-related changes that contribute to the development of this form of vasculitis. Consequently, defining the disease pathogenesis requires us to explore the mechanisms of immune ageing.

During immunosenescence, a progressive impairment of adaptive immunity is observed, driven by thymic involution and disruption of the T-cell compartment. The output of naïve T cells declines, while there is a relative expansion of long-lived, senescent memory T-cell populations. Concurrently, older individuals develop a state of “inflammaging”, characterized by chronic, low-grade systemic inflammation [1,2].

The accumulation of senescent cells sustains chronic inflammation. Despite permanent cell-cycle arrest, these cells remain metabolically active and adopt the senescence-associated secretory phenotype (SASP) [3]. This phenotype is characterized by continuous secretion of a broad range of pro-inflammatory mediators, including interleukin-6 (IL-6), tumor necrosis factor-alpha (TNF-α), and interleukin-8 (IL-8) [4]. Although SASP is considered an adaptive response to cumulative antigenic and environmental stress, it becomes maladaptive once compensatory mechanisms are overwhelmed. When uncontrolled, this chronic inflammatory state disrupts immune tolerance and creates a permissive environment for autoimmune and autoinflammatory diseases, including PMR and GCA [2,3,4,5].

Understanding immunosenescence and inflammaging is important when selecting therapy in older adults. Conventional non-selective immunosuppression, primarily based on long-term GC therapy, is associated with an increased risk of infectious and metabolic complications [6,7]. By affecting senescent cell activity, these drugs can reduce excessive inflammation more selectively. Importantly, it avoids profound systemic immunosuppression, which is particularly relevant in older patients with multiple comorbidities.

### 1.1. Pathophysiology of GCA

GCA is a granulomatous vasculitis whose pathogenesis is driven by a pathological interaction between immune cells and the vascular wall [8]. The inflammatory cascade typically begins in the tunica adventitia, where resident dendritic cells (DCs) act as vascular sentinels, detecting danger signals (PAMPs and DAMPs) primarily via Toll-like receptors (TLRs). A key defect in GCA involves impaired immune checkpoint regulation, as these DCs exhibit insufficient expression of programmed death-ligand 1 (PD-L1). Loss of this inhibitory signal results in uncontrolled T-cell activation and their massive recruitment into the perivascular space [8,9].

Acting as pathological costimulators, these dendritic cells drive naive T cells to polarize into two main distinct subsets: Th1 and Th17. Driven by the interleukin-12 (IL-12) axis, Th1 cells produce large amounts of interferon-gamma (IFN-γ), a cytokine pathway notably resistant to conventional GC therapy. In parallel, Th17 cells—induced by IL-6 and IL-23—produce interleukin-17 (IL-17), which promotes recruitment of macrophages and neutrophils [8,10]. Regulatory T cells are also functionally impaired, losing their suppressive capacity within the highly inflammatory vascular microenvironment. As inflammation becomes chronic, infiltrating immune cells organize into granulomatous structures and formations resembling arterial tertiary lymphoid organs (ATLOs). These structures, composed of T cells, tissue-resident B cells, and high endothelial venules (HEVs), serve as local sites of sustained immune activation and self-amplification of inflammation [8,11].

Within these inflammatory infiltrates, activated macrophages become a major source of interleukin-6 (IL-6) and interleukin-1β (IL-1β) [12]. Supported by neutrophils that deploy extracellular traps (NETs) and reactive oxygen species (ROSs), these giant cells unleash massive quantities of matrix metalloproteinases, primarily MMP-9 and MMP-2. This continuous enzymatic degradation fragments the internal elastic lamina, disrupting the architecture of the tunica media [8,13,14]. This is followed by pathological vascular remodeling, which constitutes the principal driver of clinical manifestations. Damage to the elastic lamina initiates signaling pathways that promote the migration of vascular smooth muscle cells (VSMCs) from the media into the tunica intima. Under the influence of local growth factors, including platelet-derived growth factor (PDGF), vascular endothelial growth factor (VEGF), and endothelin-1, VSMCs undergo a phenotypic transition into myofibroblasts. Their proliferation and extracellular matrix deposition drive the development of rapid intimal hyperplasia [8,14,15,16]. As this process progressively occludes the vessel lumen, it leads to the most severe complications of GCA, such as limb claudication and permanent vision loss. Because these intricate cytokine and proliferative networks depend heavily on JAK-STAT signaling and rapid de novo nucleotide synthesis, targeting them with small-molecule drugs offers a highly rational and precise therapeutic strategy [13,14].

### 1.2. Pathophysiology of PMR

Although clinically defined by pain and stiffness in the shoulder and pelvic girdles, PMR is fundamentally a systemic inflammatory syndrome [17]. At the molecular level, the disease is driven by age-related immune dysregulation, which specifically targets the synovial membranes and periarticular bursae [17,18]. The inflammatory cascade in PMR begins with the innate immune system, activating tissue-resident dendritic cells and macrophages [17]. Upregulated expression of Toll-like receptors on these cells drives their rapid maturation and intense antigen presentation [17,19]. However, unlike the destructive vasculitis seen in GCA, the microenvironment in PMR takes a different path: it is defined by neovascularization and synovial proliferation, triggered by a local, macrophage-driven production of vascular endothelial growth factor (VEGF) [17,20].

A hallmark of PMR pathogenesis is the dysregulation of the adaptive immune system. A marked expansion of senescent T cells is observed in both the circulation and the affected tissues of these patients [17]. These senescent cells lose the expression of the CD28 costimulatory molecule and instead aberrantly express the activating NKG2D receptor, which is typically present at lower levels on healthy cytotoxic cells [17,21]. At the same time, the pro-inflammatory environment creates a significant imbalance among T helper cells: regulatory T cells (Tregs) decrease, while highly active, harmful Th17 cells multiply [17,22]. Another key finding is the redistribution of B cells. During active disease, B-cell levels in the blood drop as C-reactive protein (CRP) and B-cell-activating factor (BAFF) levels rise. This pattern strongly suggests that B cells are actively migrating into the inflamed tissues around the joints [17,23].

The main driver of PMR’s systemic symptoms—such as fever, weight loss, and a rapid elevation in acute-phase proteins—is the production of IL-6 [17]. Given the involvement of this cytokine in PMR pathogenesis, targeted small-molecule inhibitors may offer a plausible therapeutic strategy. Upon binding to its receptor, IL-6 signals intracellularly primarily through the JAK-STAT pathway [24,25]. If we block this pathway using JAK inhibitors (JAKis), the cells stop responding to IL-6. This reduces the systemic inflammation and might help patients avoid toxic, high-dose GCs [24,26].

Furthermore, overactive Th17 cells and production of acute-phase proteins create a metabolic demand for de novo nucleic acid synthesis [27]. Conventional agents, often referred to as classic synthetic disease-modifying drugs (csDMARDs), exert their effects by interfering with pyrimidine and purine synthesis. Specifically, these compounds inhibit key enzymes within nucleotide biosynthetic pathways: mycophenolic acid inhibits inosine monophosphate dehydrogenase (IMPDH), leflunomide targets dihydroorotate dehydrogenase (DHODH), and methotrexate modulates the adenosine and folate pathways [27,28]. Fundamentally, these therapies appear to share a common mechanistic pathway: limiting the cellular availability of essential purines and pyrimidines. Through this metabolic restriction, csDMARDs are thought to preferentially constrain rapidly proliferating, autoreactive effector cells. Consequently, this helps to mitigate local joint inflammation and may facilitate a return to physiological immune balance [27,28].

## 2. Methodology: Literature Search Strategy

With the aim of providing a balanced and thorough appraisal of the available evidence, a narrative literature review was conducted. Searches were performed across PubMed, Scopus, and Web of Science, targeting peer-reviewed articles published in English. Key search terms included “polymyalgia rheumatica,” “giant cell arteritis,” “immunosenescence,” “Janus kinase inhibitors,” “csDMARDs,” and “steroid-sparing therapies.”

The selection strategy prioritised phase II and III randomised controlled trials (RCTs), meta-analyses, and large real-world observational cohorts as primary sources for evaluating clinical efficacy and safety. Mechanistic and in vitro studies were incorporated where they helped contextualise the pathophysiological framework. Case reports, small open-label trials, and retrospective series were considered where higher-level evidence was absent—principally to capture emerging therapies or specific toxicity signals- with their methodological limitations acknowledged throughout the text.

## 3. Small-Molecule Drugs—Characteristics and Mechanisms of Action

The landscape of contemporary rheumatology and clinical immunology is gradually evolving, reflecting the broader application of targeted small-molecule therapies. These agents are chemically synthesized, low-molecular-weight organic compounds (conventionally < 500 Da) that can most often be administered orally. Owing to their size, they can penetrate cells and interact with intracellular targets, thereby modulating key proteins and downstream molecular pathways [29,30]. These molecules represent diverse chemical classes and exhibit substantial differences in their mechanisms of action, targeting and modulating various inflammatory pathways—Table 1.

**Figure 1:**
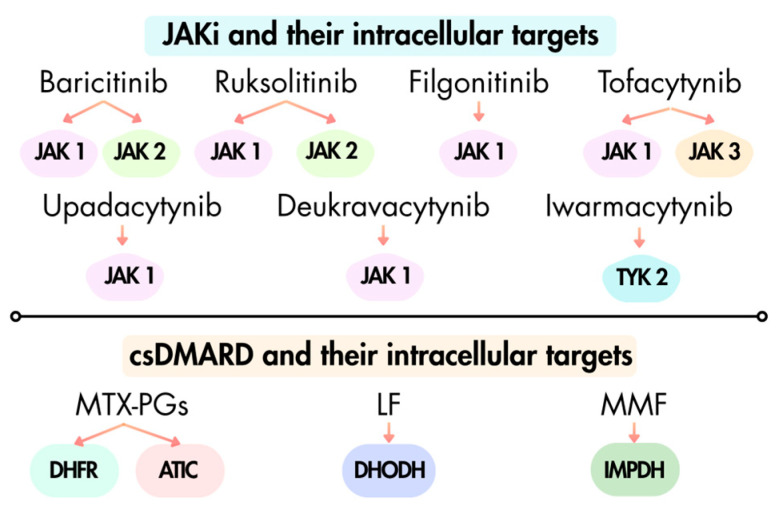
Schematic representation of intracellular targets and mechanisms of action of small-molecule drugs in immune signaling.

**Figure 2:**
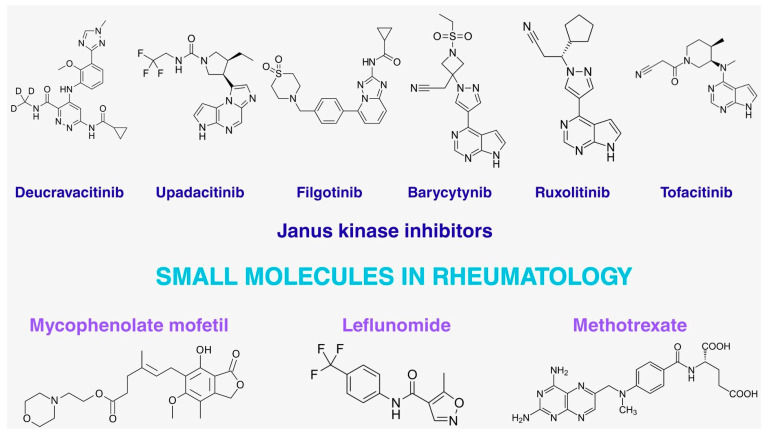
Chemical structures of analyzed small molecules.

In rheumatological nomenclature, small-molecule agents are categorized as tsDMARDs (targeted therapies based on small-molecule drugs) or csDMARDs. In contrast to bDMARDs (biologic disease-modifying antirheumatic drugs), which act predominantly in the extracellular space by neutralizing soluble mediators or blocking surface receptors, small-molecule drugs typically operate intracellularly and exhibit diverse selectivity profiles. Certain classes can modulate signaling downstream of multiple cytokine receptors, thereby attenuating immune activation and expanding treatment options for refractory autoimmune disease. Their use in older patients may improve outcomes by enabling steroid-sparing strategies and reducing the need for chronic GC therapy. This group includes both newer preparations, such as JAKi, and established conventional compounds used broadly in rheumatology (methotrexate, leflunomide, mycophenolate mofetil). The intracellular targets and specific molecular pathways modulated by the discussed conventional and targeted synthetic DMARDs are schematically illustrated in Figure 1 [29,30,31]. The structure of the small molecules discussed in the text is shown in Figure 2.

### 3.1. Janus Kinase Inhibitors

Janus kinase inhibitors exemplify targeted pharmacotherapy and constitute a class of small-molecule drugs with a well-defined, mechanism-based mode of action. Their activity relies on direct modulation of the JAK–STAT pathway, which serves as a key signaling conduit between cytokine receptors at the cell surface and the cell nucleus [32]. By blocking intracellular signal transduction, these molecules can modulate signaling downstream of multiple cytokine receptors involved in chronic inflammation.

Understanding the pharmacodynamics of this drug class requires consideration of the architecture of the physiological JAK–STAT pathway. The Janus kinase family comprises four non-receptor intracellular tyrosine kinases: JAK1, JAK2, JAK3, and TYK2 [32]. These proteins share a highly conserved domain organization, including a FERM domain (mediating association with cytokine receptors), an SH2-like domain, a pseudokinase domain (JH2), and a catalytic kinase domain (JH1) located at the C-terminus [33]. The JH2 domain warrants particular emphasis, as it functions as an important negative regulator by allosterically restraining the activity of the adjacent catalytic domain [34].

Physiological activation of the cascade is initiated by binding of a cytokine (e.g., interleukin-6, type I and II interferons, IL-12, or IL-23) to its membrane receptor. This induces receptor dimerization and promotes ATP-dependent phosphorylation and activation of receptor-associated JAK kinases. Activated JAKs recruit and phosphorylate STAT proteins (signal transducers and activators of transcription) [35]. Phosphorylated STAT monomers then form homo- or heterodimers and translocate to the nucleus, where they act as transcription factors that modulate target gene expression [36].

At the molecular level, JAK inhibitors interfere with this pathway through distinct mechanisms. Most agents in this class competitively inhibit the ATP-binding site within the catalytic JH1 domain [37]. This abrogates kinase activity, prevents STAT phosphorylation, and consequently inhibits transmission of inflammatory signals to the nucleus [38]. Advances in drug design have also enabled the development of molecules with allosteric activity. Deucravacitinib represents this approach by selectively binding and stabilizing the JH2 domain of TYK2, without direct interaction with the ATP-binding site [39].

Because cytokine receptor signaling typically requires JAK kinase pairs, the relative affinity of individual inhibitors for specific isoforms shapes their pharmacodynamic profiles. Available data indicate that tofacitinib exhibits preferential inhibition of JAK1 and JAK3 (with lower affinity for JAK2 and TYK2), whereas baricitinib and ruxolitinib primarily target JAK1 and JAK2 [40]. Upadacitinib and filgotinib, in turn, demonstrate higher selectivity for JAK1 [40].

Isoform selectivity influences the breadth of immune-axis modulation. In vitro studies suggest that, in rheumatoid arthritis, therapeutic efficacy is largely associated with inhibition of JAK1-dependent signaling, including interferon-alpha/pSTAT5 and interleukin-6/pSTAT1 axes [41]. In parallel, inhibition of JAK2-dependent pathways may contribute through modulation of platelet activity involved in synovial inflammation, whereas JAK3 blockade appears important for suppressing lymphocyte proliferation driven by IL-7 and IL-15 [42]. From a translational standpoint, however, the safety profile of JAK inhibitors in routine clinical practice is determined less by theoretical molecular selectivity and more by the administered dose and the patient’s demographic and clinical characteristics, including comorbidity burden [42,43].

In light of their broad immunomodulatory effects, the clinical application of JAK inhibitors necessitates rigorous patient screening based on comorbidity profiles. According to recent European Alliance of Associations for Rheumatology (EULAR) guidelines, several critical warnings and contraindications dictate their use. JAK inhibitors are contraindicated in the presence of severe active or recurrent infections, including tuberculosis and opportunistic pathogens, and are associated with a dose-dependent risk of herpes zoster. Furthermore, these agents warrant extreme caution in patients with a current or past history of malignancies; notable imbalances in lung cancer incidence have been observed in populations with high cardiovascular risk and a history of smoking. Severe organ dysfunction constitutes an absolute contraindication, specifically decompensated advanced chronic liver disease (e.g., Child–Pugh score ≥ 9) and severe renal impairment, with agents such as baricitinib, filgotinib, and upadacitinib being strictly contraindicated at a creatinine clearance of <15 mL/min. Additionally, a history of arterial or venous thromboembolic events requires a stringent risk-benefit assessment due to a documented dose-dependent thromboembolic risk. Finally, JAK therapy is contraindicated during pregnancy and lactation, as well as in conjunction with live vaccines [44].

### 3.2. Leflunomide

Leflunomide (LF), a csDMARD, is an immunomodulatory agent whose biological activity depends on conversion to its active metabolite, teriflunomide. Its primary mechanism of action involves selective inhibition of dihydroorotate dehydrogenase (DHODH), an enzyme located in the inner mitochondrial membrane that catalyzes the rate-limiting step of de novo pyrimidine nucleotide biosynthesis (conversion of dihydroorotate to orotate) [45]. While resting cells can satisfy basal pyrimidine requirements via salvage pathways, immune activation—particularly clonal expansion of autoreactive lymphocytes and activation of innate immune cells (monocytes, macrophages)—is associated with a marked increase in nucleotide demand that relies largely on de novo synthesis [28]. By depleting intracellular pyrimidine pools, LF exerts antiproliferative and immunosuppressive effects within the inflammatory environment, thereby limiting the expansion of effector cells that sustain autoimmunity. This may secondarily reduce immunoglobulin production and modulate cytokine output toward a less pro-inflammatory profile. Moreover, recent basic research reports suggest additional antiviral and antineoplastic potential of LF and its derivatives [28].

Beyond DHODH inhibition, LF displays pleiotropic activity, including suppression of intracellular tyrosine kinases involved in immune signal transduction [46]. Inhibition of tyrosine phosphorylation results in multi-level attenuation of downstream signaling pathways. LF has been shown to inhibit the IL-4-driven JAK3/STAT6 axis, which interferes with immunoglobulin class switching from IgM to IgG1 [46]. In addition, LF impairs IL-2–induced T-cell proliferation and disrupts signal transduction downstream of the T-cell receptor, as demonstrated in cellular models stimulated with anti-CD3 antibodies. These DHODH-independent mechanisms complement the multidirectional immunomodulatory profile of LF and may contribute to its efficacy in both monotherapy and combination therapy in autoimmune arthritis [47].

### 3.3. Methotrexate (MTX)

Methotrexate (MTX) is a classic csDMARD whose efficacy in rheumatology stems from its penetration into the intracellular space and selective modulation of key metabolic pathways. The pleiotropic pharmacodynamic profile of the drug extends beyond its originally described folate antagonism, encompassing integrated anti-inflammatory, antiproliferative, and immunomodulatory mechanisms [48].

Cellular uptake of MTX into target cells, including lymphocytes, macrophages, and fibroblasts, occurs primarily via the reduced folate carrier 1 (RFC1/SLC19A1). In the cytosol, MTX undergoes a critical structural modification—polyglutamation catalyzed by folylpolyglutamate synthetase (FPGS) [49]. The addition of glutamate residues increases the negative charge of methotrexate polyglutamates (MTX-PGs), limiting their efflux and thereby promoting intracellular retention. MTX-PGs exhibit higher affinity for several target enzymes than the parent compound, which supports sustained pharmacological activity despite fluctuations in serum MTX concentrations [49].

A key folate-dependent mechanism of MTX-PGs is competitive inhibition of dihydrofolate reductase (DHFR). Depletion of intracellular tetrahydrofolate pools impairs de novo purine and pyrimidine synthesis and, consequently, nucleic acid replication. In an inflammatory microenvironment, this can exert a cytostatic effect that limits clonal expansion of proliferating autoreactive T and B lymphocytes [48].

Independent of folate availability, an important axis of MTX anti-inflammatory activity involves modulation of adenosine metabolism. By inhibiting AICAR transformylase (ATIC), MTX promotes intracellular accumulation of 5-aminoimidazole-4-carboxamide ribonucleotide (AICAR) and related metabolites [49]. These compounds inhibit AMP deaminase and adenosine deaminase (ADA), reducing intracellular adenosine catabolism and, in cooperation with surface ectoenzymes CD39 and CD73, facilitating adenosine export to the extracellular space. Extracellular adenosine, acting as an endogenous autacoid, binds to A2A (ADORA2A) and A3 (ADORA3) receptors expressed on immunocompetent cells. Activation of these receptors can limit neutrophil chemotaxis and attenuate the production of pro-inflammatory cytokines, including IL-1β, TNF-α, IL-6, and IL-12, as well as matrix metalloproteinases (MMPs) by macrophages and lymphocytes [50].

Additional molecular effects of MTX have also been described. MTX administration has been reported to increase reactive oxygen species (ROSs), which may sensitize activated T lymphocytes to apoptosis via a c-Jun N-terminal kinase (JNK)-dependent pathway, thereby facilitating elimination of cells sustaining inflammation [51]. Moreover, through effects on folate-dependent one-carbon metabolism, MTX may influence the methionine cycle. Reduced availability of 5-methyltetrahydrofolate may decrease intracellular S-adenosylmethionine (SAM), the principal methyl group donor, potentially contributing to global hypomethylation of DNA and proteins in immune and endothelial cells. This, in turn, has been associated with reduced expression of adhesion molecules (including ICAM-1 and VCAM-1), which may impair leukocyte migration into the perivascular compartment of inflamed vessels [52].

### 3.4. Mycophenolate Mofetil (MMF)

Mycophenolate mofetil (MMF) is a prodrug that undergoes rapid in vivo conversion to pharmacologically active mycophenolic acid (MPA). The immunosuppressive activity of MPA is primarily mediated by selective, reversible, and non-competitive inhibition of inosine monophosphate dehydrogenase (IMPDH), the key rate-limiting enzyme in de novo guanosine nucleotide biosynthesis [53]. Because activated T and B lymphocytes display a marked dependence on this pathway, MPA can exert a selective cytostatic effect in these populations. This selectivity may be further supported by the higher affinity of MPA for the type II isoform of IMPDH, which is upregulated in proliferating lymphoid cells [53].

In the context of the pathomechanisms of GCA and PMR, the activity profile of MMF/MPA may extend beyond cytostasis to mechanisms relevant to systemic vasculitides. First, in addition to inhibiting proliferation, MPA has been reported to promote apoptosis in subsets of activated T lymphocytes, potentially facilitating elimination of clones sustaining chronic immune activation [54]. Secondly, depletion of intracellular guanosine nucleotides may impair protein glycosylation, which can reduce the surface expression of selected adhesion molecules. In turn, this may limit recruitment and transmigration of pathogenic monocytes and lymphocytes from the vascular lumen into perivascular inflammatory sites [54].

An additional aspect of MMF/MPA activity, potentially relevant to ischemic complications in GCA, is its effect on vascular remodeling. Progressive arterial occlusion in GCA is driven in part by pathological intimal hyperplasia mediated by vascular smooth muscle cells. MPA at therapeutic concentrations has been shown to inhibit growth factor-stimulated smooth muscle cell proliferation, which may translate into attenuation of occlusive lesion development [55]. This putative vascular effect may be complemented by modulation of local oxidative/nitrosative stress. Because GTP serves as a precursor in BH4 biosynthesis, MMF-associated reductions in intracellular GTP may decrease BH4 availability, an essential cofactor for iNOS. This could reduce macrophage NO production and consequently may limit peroxynitrite formation implicated in vascular injury [56].

## 4. Clinical Efficacy of Small-Molecule Drugs in GCA and PR

### 4.1. The Use of Janus Kinase Inhibitors in GCA

Initial clinical evidence for the efficacy of JAKi in GCA emerged from prospective pilot studies and real-world retrospective analyses, primarily involving patients with relapsing or refractory disease. In a Swedish case series of 15 GCA patients treated with baricitinib or tofacitinib (due to inadequate GC response or unsuitability for IL-6 blockade), reductions in CRP, erythrocyte sedimentation rate (ESR), and daily prednisone doses were observed within 3 to 6 months. Notably, no relapses occurred during a median follow-up of 19 months [24].

A larger multi-center real-world study evaluated 35 patients with relapsing GCA treated with baricitinib, tofacitinib, or upadacitinib. The majority of these patients had refractory disease, having previously failed conventional synthetic immunosuppressants (63%) and biologic agents like tocilizumab (86%). After a median follow-up of 11 months, 57% of patients achieved clinical remission and 46% achieved complete remission. Furthermore, the median daily prednisone dose decreased significantly from 16.2 mg to 5 mg, with 20% of patients discontinuing GCs entirely [57].

The most compelling evidence for JAKi in GCA comes from the SELECT-GCA Phase 3 randomized, double-blind, placebo-controlled trial evaluating upadacitinib, a selective JAK1 inhibitor. The trial randomized patients to receive either upadacitinib 15 mg daily or 7.5 mg daily (both combined with a 26-week GC taper) versus a placebo combined with a 52-week GC taper [58,59].

Although the SELECT-GCA trial met its primary endpoint—demonstrating sustained remission in 46% of patients on 15 mg upadacitinib versus 29% on placebo—the broader clinical picture requires careful appraisal. With over half of the 15 mg cohort failing to maintain remission, and the lower 7.5 mg dose failing to demonstrate any significant superiority over placebo, the data reveal a substantial efficacy gap [58,59]. Consequently, while regulatory approval is highly anticipated, this partial response illustrates the difficulty of achieving comprehensive disease control through the inhibition of a specific signalling axis in a highly heterogeneous condition [60].

JAK inhibitors are generally well-tolerated in the older adult GCA population, though their safety profile requires careful monitoring. In observational studies, adverse events leading to drug discontinuation occurred in a minority of patients, including cases of significant liver enzyme elevation, dyspnea, and isolated reports of malignancy [57].

Infections are the most commonly reported adverse events. Observational cohorts have noted serious infections, including *Enterococcus faecalis* bacteremia and *Aspergillus fumigatus* pneumonia, which required antibiotic or antifungal treatment and temporary interruption of the JAKi [24,59]. In the Phase 3 SELECT-GCA trial, patients taking upadacitinib 15 mg daily showed a higher risk for opportunistic infections, herpes zoster, and non-melanoma skin cancer compared to the placebo group, though there was no increased risk observed for major adverse cardiovascular events (MACEs) or venous thromboembolism [58,59].

Current evidence suggests that JAK inhibitors—particularly upadacitinib, baricitinib, and tofacitinib—represent a compelling, oral, targeted therapy for GCA. By disrupting the primary cytokine pathways driving vascular inflammation, they offer a valuable steroid-sparing strategy, even for patients who have proven refractory to established biologics such as tocilizumab.

Despite this promise, there is a clear discrepancy between the theoretical elegance of JAK-STAT inhibition and the actual clinical remission rates of approximately 50%. This limit in therapeutic success suggests that addressing inflammation alone may not be sufficient, a challenge that likely stems from two key factors. On the one hand, while JAK inhibitors successfully quieten cytokine signalling, their impact on downstream structural vascular changes appears minimal. Pathological remodelling can reach a stage of autonomous progression where intimal hyperplasia and cell migration no longer depend solely on the initial inflammatory trigger. Once this vascular damage is established, purely anti-inflammatory molecules may lack the capacity to reverse it. On the other hand, senescent immune cells complicate matters further. Because they continuously release a broad spectrum of mediators despite cell-cycle arrest, they may easily exploit alternative, non-JAK-dependent pathways. This redundancy would naturally render them partially resistant to such highly targeted suppression [57,58,59,60].

### 4.2. Conventional Synthetic Disease-Modifying Antirheumatic Drugs in GCA

MTX is the most studied and widely utilized csDMARD for the treatment of GCA, though its overall efficacy is considered modest. This limited therapeutic success likely stems from MTX’s mechanism of action. Conventional DMARDs restrict nucleotide availability, preferentially targeting rapidly proliferating cells. However, GCA is driven by immunosenescence; key pathogenic populations—such as senescent T cells and macrophages—reside in permanent cell-cycle arrest while remaining metabolically active and secreting pro-inflammatory mediators. Consequently, purely anti-proliferative strategies are inherently limited in their capacity to resolve this inflammatory microenvironment. While individual, underpowered randomized controlled trials (RCTs) using relatively low doses of MTX yielded mixed or negative results, a meta-analysis of three placebo-controlled trials demonstrated a benefit. The analysis revealed that MTX (typically dosed at 10–15 mg/week) lowers the relapse rate (hazard ratio 0.65), reduces the cumulative GC dose by a mean of 842 mg at 48 weeks, and yields a higher rate of GC-free remission (hazard ratio 2.8) [61,62].

Despite these benefits, the number needed to treat to prevent a single cranial relapse remains high (ten patients), and MTX does not appear to significantly diminish overall GC-related adverse events [61,62]. Consequently, the British Society for Rheumatology (BSR), the European Alliance of Associations for Rheumatology (EULAR), and the American College of Rheumatology (ACR) offer a conditional recommendation for MTX. It is generally advised as an adjunctive therapy, in combination with a GC taper, primarily for patients who are at a high risk of GC toxicity, those who experience relapses, or those who cannot access biologic therapies like tocilizumab [62].

Beyond methotrexate, clinical data supporting other csDMARDs is generally restricted to small trials, case series, and retrospective cohorts, making their role in standard GCA management poorly defined.

Azathioprine: Evidence for azathioprine is primarily derived from a single, small, double-blind, randomized controlled trial of 31 patients with GCA or PMR. In this specific cohort, azathioprine demonstrated a significant reduction in the mean GC requirement over one year of therapy, but broader validation is lacking [63].

Leflunomide: Experience with leflunomide is similarly sparse, with one small study involving 12 patients with PMR and 11 with GCA pointing out a potential efficacy as a GC-sparing agent, though this requires confirmation in larger RCTs [64].

Mycophenolate Mofetil (MMF): A retrospective cohort study evaluating 37 patients with large-vessel vasculitis GCA prescribed mycophenolate derivatives reported relapse rates of 16.2% at 1 year and 27% at 2 years. However, its benefit as a reliable GC-sparing agent remains limited [65].

Cyclophosphamide: Unlike the other agents, cyclophosphamide has shown an ability to control highly refractory disease. A French study of 103 patients with GC-dependent or GC-resistant GCA demonstrated that over 50% of patients achieved long-term remission, successfully reduced their GCs, and showed regression of vascular activity on PET (Positron Emission Tomography) imaging. However, the severe adverse event profile and high toxicity associated with cyclophosphamide generally preclude its consideration as a standard GC-sparing therapy [62,66].

Several other conventional immunosuppressants have been evaluated and effectively ruled out for GCA management. Randomized and open-label trials assessing hydroxychloroquine, cyclosporine A, and dapsone all failed to demonstrate any beneficial effects or significant GC-sparing capabilities in patients with GCA [62].

In summary, outside of methotrexate, there is insufficient evidence or an unacceptable safety profile to recommend the routine use of other oral conventional immunosuppressive agents in the management of GCA.

### 4.3. The Use of Janus Kinase Inhibitors in PMR

Tofacitinib, an inhibitor of JAK1 and JAK3, has been evaluated in several contexts for its efficacy as a GC-sparing agent in PMR. In a protocolized, two-stage minimax design study, researchers evaluated 14 patients with highly active PMR receiving 10 mg/day of tofacitinib alongside a tapering dose of prednisone, starting at 15 mg/day. At week 24, 85.7% of the patients achieved a remission response, defined as sustained low disease activity with GC independence. This treatment resulted in significant improvements in quality of life and sustained reductions in disease activity scores without relapses during the extension phase [26].

Furthermore, a Phase II, randomized, open-label trial by Ma et al. compared tofacitinib (5 mg twice daily) to a tapering regimen of GCs in 76 newly diagnosed PMR patients. The study found no statistically significant difference in outcomes between the two groups over 24 weeks. Notably, 100% of patients in both groups achieved a PMR Activity Scale (PMR-AS) of less than 10 at weeks 12 and 24, demonstrating that tofacitinib monotherapy offers similar clinical benefits to traditional GC treatment [67].

Additionally, a retrospective analysis compared 15 female PMR patients treated with JAK inhibitors, predominantly tofacitinib, against 15 patients treated with csDMARDs, specifically methotrexate. While both groups showed similar improvements in inflammatory markers, the JAK inhibitor group required significantly lower GC doses at 3 and 6 months, highlighting its utility as an effective GC reducer [68].

Baricitinib, a JAK1 and JAK2 inhibitor, has also demonstrated efficacy in achieving GC-free remission. The Phase II BACHELOR study randomized early PMR patients to receive either 4 mg/day of baricitinib or a placebo, without the use of oral GCs (though optional subdeltoid GC injections at weeks 1 and 4 were permitted). By week 12, 77.7% (14 of 18) of patients in the baricitinib group achieved sustained low disease activity (defined as a CRP PMR-AS of 10 or less) compared to only 13.3% (2 of 15) in the placebo group. Furthermore, stepping the dosage down to 2 mg/day from weeks 12 to 24 successfully maintained treatment efficacy, with no flares observed at week 36 despite stopping the medication at week 24 [69,70]. Following these positive results, the ongoing Phase III JAK-SPARE1 study is currently evaluating baricitinib’s ability to achieve GC-free remission after 16 weeks of treatment in newly diagnosed PMR patients [70].

While early phase trials and retrospective cohorts indicate that JAK inhibitors—notably tofacitinib and baricitinib—hold considerable steroid-sparing promise, describing them as universally highly effective warrants a degree of caution.

The current evidence base is largely drawn from smaller cohorts with relatively brief follow-up periods. Furthermore, because the SASP is regulated through multiple overlapping pathways, JAK inhibition alone may not provide adequate disease control in all patients. However, larger studies with longer follow-up are still needed to better define the efficacy and long-term safety of these agents in older patients with PMR [70].

### 4.4. The Use of Conventional Synthetic DMARDs in PMR

MTX is the most widely recommended and utilized csDMARD for PMR. Current guidelines conditionally recommend the early introduction of MTX for patients at a high risk of relapse or those with comorbidities, such as diabetes or osteoporosis, where GC toxicity is a major concern [70,71,72].

A randomized trial by Caporalli et al. demonstrated that patients taking 10 mg/week of MTX alongside a prednisone taper required significantly lower median cumulative prednisone doses compared to a placebo group [73]. Similarly, Ferraccioli et al. found that intramuscular MTX lowered cumulative prednisone doses and stabilized bone mineral density over a 12-month period [74]. Despite these positive findings, the efficacy of MTX is not universally supported. A trial by Van der Veen et al. found no significant differences between MTX and placebo groups regarding time to remission, relapse frequency, or total prednisone dose [75].

Similar to GCA, the anti-proliferative nature of MTX makes it ineffective against senescent immune cells, explaining the suboptimal steroid-sparing results in PMR [74,75].

Beyond methotrexate, leflunomide has shown promise, particularly for difficult-to-treat or refractory PMR cases, although it requires further validation through prospective studies [64,70]. Retrospective studies by Diamantopoulos et al. and Adizie et al. indicated that a starting dose of 10 mg/day of leflunomide was well tolerated and facilitated the tapering or complete discontinuation of GCs in patients who had failed optimal MTX therapy [64,76]. A comparative study by Vinicki et al. reported that PMR patients treated with leflunomide experienced a higher rate of GC withdrawal (72%) compared to those on MTX (39%). Furthermore, the median time to prednisone discontinuation was substantially shorter for the leflunomide cohort, measured at 4.7 months versus 31.8 months for the MTX group [77]. Leflunomide and MTX are currently being compared in the Phase III STERLING-PMR trial for patients who have experienced at least one relapse [70].

Finally, data on azathioprine in the context of PMR are extremely limited. A small double-blind randomized controlled trial suggested a GC-sparing effect at the one-year mark, but overall efficacy is not strongly supported, and it is not currently included in major management recommendations for PMR [63].

Therefore, as a practical therapeutic solution across both PMR and GCA, JAK inhibitors (like tofacitinib or baricitinib) should be prioritized as the primary steroid-sparing agents owing to their robust clinical trial data and better outcomes. Conventional csDMARDs should be strictly reserved for patients lacking access to JAK inhibitors or those presenting with clear contraindications.

### 4.5. The Use of Clofutriben (SPI-62) in PMR

The clinical utility of clofutriben, a selective HSD-1 inhibitor (11β-hydroxysteroid dehydrogenase type 1 inhibitor) previously evaluated for safety and pharmacokinetics in healthy volunteers [78], was recently evaluated in a proof-of-concept and dose-range finding trial in PMR. The study enrolled adult patients with established PMR, who were successfully stabilized on a standard dose of 10 mg/day of prednisolone. During the trial, patients were administered a daily 6 mg dose of clofutriben co-administered with varying increased doses of prednisolone (10, 15, 20, or 30 mg/day) to observe how HSD-1 inhibition shifted the dose–response relationship for both the disease control and the toxicity of the steroid [79].

The trial demonstrated that adding clofutriben to the baseline 10 mg/day prednisolone dose significantly reduced the active intracellular GC exposures, which led to an expected pharmacological reduction in efficacy at the baseline dose. Patients in this group experienced clinical relapses, worsened PMR symptoms (measured by numeric rating scales for pain, stiffness, and fatigue), increased physical disability, and elevated acute phase inflammatory biomarkers like ESR and CRP [79]. However, the study successfully proved that by increasing the co-administered prednisolone dose to 20 mg/day or 30 mg/day alongside clofutriben, PMR disease control and clinical efficacy were fully restored to the levels originally achieved by the 10 mg/day prednisolone monotherapy [79].

A notable clinical finding for PMR management was clofutriben’s ability to diminish and reverse GC-associated morbidities, building on prior evidence that HSD-1 inhibition can minimize prednisolone-induced adverse effects [80], even when the co-administered prednisolone doses were doubled or tripled. Across all evaluated regimens, clofutriben yielded favorable improvements in multiple toxicity biomarkers. This included the normalization of bone turnover markers (osteocalcin, PINP [procollagen type I N-terminal propeptide], and CTX [C-terminal telopeptide of type I collagen]), directly countering the known role of HSD-1 in GC-associated bone loss [81]. It also improved lipid metabolism with the normalization of elevated triglycerides, and improved hypercoagulability parameters (Activated Partial Thromboplastin Time). Furthermore, clofutriben rapidly reversed hypothalamic–pituitary–adrenal (HPA) axis suppression, shifting patients from a high risk of adrenal insufficiency to a reduced or low risk based on morning serum cortisol and ACTH (adrenocorticotropic hormone) levels [79].

Overall, the combination of clofutriben and prednisolone was generally well tolerated. Treatment-emergent adverse events were mostly mild to moderate, with no serious adverse events reported. The most notable effects included a numerical increase in mild, non-serious upper respiratory infections and anticipated increases in androgens (testosterone), though no clinical symptoms of hyperandrogenism were observed [79].

## 5. Geriatric Considerations and Future Development Perspectives

PMR and GCA are age-associated inflammatory diseases with incidence peaking in the seventh and eighth decades of life. Accordingly, their management is closely shaped by physiological decline in organ function, multimorbidity, and polypharmacy in older adults [82,83,84].

GCs remain the cornerstone of therapy, but prolonged exposure in older patients carries a substantial morbidity burden. Age-related muscle loss is compounded by steroid-associated myopathy and sarcopenia, increasing fall and frailty risks. Concurrently, GC therapy accelerates the deterioration of bone microarchitecture, exacerbating osteoporosis and fracture risk. These factors necessitate effective GC-sparing strategies, including the rational use of immunomodulatory therapies [85,86]. However, therapeutic immune modulation with small-molecule agents must be weighed against immunosenescence; Janus kinase inhibitors (JAKi) can attenuate pathogenic inflammation but may also impair host defense, leading to dose-dependent viral reactivation (e.g., Varicella–Zoster Virus) [57,87].

The implementation of conventional and targeted synthetic DMARDs requires careful attention to pharmacokinetic (PK) and pharmacodynamic (PD) changes. Based on current consensus, dose adjustments for JAKi must be individualized. Chronological age alone does not dictate dosing; rather, modifications must account for cumulative comorbidities, frailty, and physiological decline. A progressive decline in glomerular filtration rate (eGFR) necessitates dose reductions to prevent drug accumulation and toxicity. Baricitinib is highly renally excreted (>66–70%) and requires dosage reduction in patients with kidney disease. Methotrexate (MTX) also mandates strict renal dosing to avoid bone marrow suppression [44,85,87].

Similarly, hepatic metabolism and drug–drug interactions warrant heightened vigilance, especially adjustments of dosage are necessary for agents metabolized via cytochrome P450 pathways, including selected Janus kinase inhibitors (JAKi) such as tofacitinib and upadacitinib. In patients with PMR or GCA who frequently receive multiple cardiovascular, metabolic, and neuroactive medications, competitive CYP3A4 inhibition or induction may occur (Table 2). Clinically relevant interactions can increase systemic exposure and contribute to serious adverse events if not recognized and managed [88,89].

### Patient-Centered Decision Making and Risk Stratification in Older Adults

Although JAK inhibitors offer an attractive oral alternative to biologic therapies and may reduce glucocorticoid exposure, their higher acquisition costs and reimbursement restrictions may limit access in routine clinical practice. In contrast, conventional synthetic DMARDs such as methotrexate remain substantially less expensive and are often more readily available. Economic considerations may therefore influence therapeutic choices, particularly among older adults with fixed incomes or dependence on caregiver support [90,91,92,93].

Medication adherence represents a crucial determinant of treatment success in the geriatric population, influenced by numerous patient-related and systemic factors. Cognitive deficits, depression, functional limitations, as well as demographic barriers and low health literacy, significantly limit compliance with complex treatment regimens. This situation is further exacerbated by clinical factors, such as suboptimal communication with the physician, polypharmacy, and logistical difficulties related to medication handling and dosing. Consequently, shared decision-making (SDM) involving both patients and their caregivers should form an integral part of treatment planning, enabling personalized care and an optimal balance between clinical benefits and treatment burden [94,95].

Frailty assessment tools such as the Clinical Frailty Scale (CFS) or Frailty Index may assist clinicians in identifying patients at increased risk of treatment-related complications. Frailty reflects reduced physiological reserve and increased vulnerability to adverse health outcomes that are not adequately captured by chronological age alone. These instruments have demonstrated prognostic value for hospitalization, disability, and mortality and may therefore provide additional support when weighing the risks and benefits of advanced immunomodulatory therapies in the oldest patients [96,97].

## 6. Monitoring Recommendations

Given the immunomodulatory potency of small-molecule drugs and the specific vulnerabilities of geriatric patients, robust clinical monitoring is imperative. Based on registry data and the ORAL Surveillance trial, initiating therapy requires a comprehensive baseline assessment to establish an individualized risk-benefit profile [44,98].

Baseline screening must exclude severe or recurrent infections (including tuberculosis) and assess historical risks for MACE, venous thromboembolism, and malignancies. Appropriate non-live vaccinations must be administered prior to treatment initiation. Severe organ dysfunction, including decompensated chronic liver disease (Child–Pugh score ≥ 9) or severe renal impairment (creatinine clearance < 15 mL/min), constitutes an absolute contraindication for agents like upadacitinib, baricitinib, and filgotinib [44].

During active treatment, routine laboratory surveillance (comprehensive metabolic panels) is essential to monitor shifting renal and hepatic function, prompting proactive dose reductions. Medication reconciliation must be performed at every follow-up to identify newly prescribed CYP3A4 modifiers. Clinicians must actively monitor for clinical adverse events, notably herpes zoster reactivation, thromboembolic complications, and opportunistic infections, while continually assessing therapeutic efficacy to facilitate safe GC tapering [44,98].

Crucially, in scenarios where a patient’s severe physical frailty, cognitive impairment, or lack of reliable social support renders adherence to complex dosing and intensive laboratory monitoring untenable, the introduction of targeted JAKi therapy should be strictly avoided [99,100]. Under these circumstances, the risk of fatal medication errors or undetected opportunistic infections and organ toxicity outweighs potential rheumatological benefits. When therapeutic burden exacerbates susceptibility to iatrogenic harm, clinical focus must shift from aggressive disease modification to pragmatic deprescribing and care simplification [100].

## 7. Future Development Perspectives

Looking ahead, therapeutic development in GCA and PMR is expected to further prioritize GC-sparing approaches, expand immunomodulatory options, and refine the role of small-molecule drugs in geriatric care. Approved IL-6 pathway—targeting therapies include tocilizumab in GCA and sarilumab in PMR, while additional approaches remain under active investigation. Emerging evidence supports the evaluation of JAK inhibition in these conditions, including clinical studies of baricitinib and tofacitinib in PMR and upadacitinib in GCA. Innovative platforms, such as antibody–drug conjugates exemplified by ABBV-154 (combining glucocorticoid receptor modulation with targeted anti-TNF activity), have also been explored with the aim of improving efficacy while limiting systemic toxicity. Collectively, these strategies may enable more individualized treatment pathways that reduce GC-related adverse effects while improving disease control [85].

## 8. Limitations

Although this review highlights what appears to be a promising shift towards targeted small-molecule therapies in PMR and GCA, the current evidence base carries a number of important limitations that warrant careful consideration.

### 8.1. Mechanistic and Efficacy Ceilings

As noted earlier, translating a strong biological rationale into clinical practice often reveals a therapeutic plateau. Even within the most rigorous trials of selective JAK1 inhibition for GCA, findings suggest that a considerable proportion of patients may not achieve or sustain remission over the long term. This raises the possibility that targeting a single intracellular signalling cascade may be insufficient to fully suppress the redundant, non-JAK-dependent pathways that senescent cells are thought to employ. Moreover, once pathological vascular remodelling and intimal hyperplasia have progressed beyond a certain point, purely anti-inflammatory interventions may lack the capacity to reverse established structural damage.

### 8.2. Methodological Constraints

Much of the clinical data underpinning the use of both csDMARDs and targeted therapies derives from underpowered pilot studies, open-label designs, and retrospective series. While recent milestone phase III trials, such as SELECT-GCA, provide welcome high-quality data, the field still largely lacks definitive, large-scale, placebo-controlled randomised trials with follow-up extending beyond 52 weeks. As a result, the long-term durability of remission and the potential for delayed adverse events remain incompletely discovered.

### 8.3. Applicability to Geriatric Cohorts

Finally, because PMR and GCA predominantly affect an older demographic, strict clinical trial inclusion criteria tend to introduce a degree of selection bias. By frequently excluding patients with severe multimorbidity, advanced frailty, or complex polypharmacy, trial cohorts rarely reflect the full reality of the clinic. Consequently, the favourable safety and efficacy profiles observed in highly controlled settings may not seamlessly translate to routine geriatric practice. In this older demographic, age-related renal decline, complex CYP3A4 drug interactions, and a heightened vulnerability to opportunistic infections or thromboembolic events may pose considerably greater clinical challenges than current trial data would suggest.

## 9. Summary

Immunosenescence fundamentally drives the pathogenesis of PMR and GCA by perpetuating a maladaptive, chronic inflammatory environment. As conventional glucocorticoid therapy exacts an unacceptable metabolic and osteoporotic toll on geriatric patients, targeted small-molecule drugs offer a pathophysiologically sound alternative. By selectively inhibiting intracellular signaling conduits—most notably the JAK-STAT pathway—and critical nucleotide biosynthesis, these advanced agents precisely attenuate immune overactivation. Targeted therapies may reduce the need for glucocorticoids and help maintain disease control while limiting treatment-related complications in older patients.

## Figures and Tables

**Table 1:** Summary of Small-Molecule Drugs in the Management of PMR and GCA.

Drug	Primary Molecular Target	Inhibited Cellular Process	Clinical Indication/Status in PMR/GCA	Main Limitation
Janus Kinase Inhibitors (JAKi)	JAK1, JAK2, JAK3, TYK2	STAT phosphorylation and nuclear transmission of inflammatory signals	Prioritized steroid-sparing agents; Upadacitinib (GCA Phase 3); Tofacitinib/Baricitinib (PMR trials)	~50% remission ceiling; cannot reverse established structural vascular damage; opportunistic infection risks
Methotrexate (MTX)	DHFR and ATIC	De novo purine and pyrimidine synthesis; adenosine catabolism	Most widely utilized csDMARD; conditional recommendation as adjunctive therapy	Modest overall efficacy; anti-proliferative approach is ineffective against senescent cells
Leflunomide (LF)	DHODH	De novo pyrimidine nucleotide biosynthesis	Potential GC-sparing agent for difficult-to-treat or refractory PMR/GCA	Evidence is largely restricted to small trials, case series, and retrospective cohorts
Mycophenolate Mofetil (MMF)	IMPDH	De novo guanosine nucleotide biosynthesis	Evaluated as adjunctive therapy for large-vessel GCA	Limited benefit as a reliable GC-sparing agent; high relapse rates in current data
Clofutriben (SPI-62)	HSD-1	Active intracellular glucocorticoid exposure	Proof-of-concept in PMR for reducing and reversing GC-induced morbidities	Requires co-administration with increased steroid doses to maintain baseline disease control

Abbreviations: ATIC, 5-aminoimidazole-4-carboxamide ribonucleotide formyltransferase; csDMARD, conventional synthetic disease-modifying antirheumatic drug; DHFR, dihydrofolate reductase; DHODH, dihydroorotate dehydrogenase; GC, glucocorticoid; GCA, giant cell arteritis; HSD-1, 11β-hydroxysteroid dehydrogenase type 1; IMPDH, inosine monophosphate dehydrogenase; JAK, Janus kinase; JAKi, Janus kinase inhibitors; LF, leflunomide; MMF, mycophenolate mofetil; MTX, methotrexate; PMR, polymyalgia rheumatica; STAT, signal transducer and activator of transcription; TYK2, tyrosine kinase 2.

**Table 2:** Clinically Significant Interactions of JAK Inhibitors with CYP3A4 Inhibitors and Inducers.

Medication Group	Commonly Used Medications	Interaction Type & Risk Level
Azole Antifungals	Ketoconazole, itraconazole, voriconazole, posaconazole, fluconazole	Strong or Moderate Inhibitor (High to Medium)
Macrolide Antibiotics	Clarithromycin, erythromycin (note: azithromycin does not inhibit CYP3A4)	Strong or Moderate Inhibitor (High to Medium)
Protease Inhibitors & Boosters	Ritonavir, cobicistat, darunavir, atazanavir, lopinavir	Strong Inhibitor (High)
Grapefruit Products	Grapefruit, grapefruit juice	Intestinal Inhibitor (High)
Non-DHP CCBs	Diltiazem, verapamil	Moderate Inhibitor (Medium)
Rifamycins	Rifampin (rifampicin), rifabutin, rifapentine	Strong Inducer (High)
Anticonvulsants	Carbamazepine, phenytoin, phenobarbital	Strong Inducer (High)
St. John’s Wort	St. John’s wort (Hypericum perforatum)	Strong Inducer (High)
NNRTI Antiretrovirals	Efavirenz, nevirapine, etravirine	Moderate Inducer (Medium)

CYP3A4—Cytochrome P450 3A4, Non-DHP CCB—Non-dihydropyridine Calcium Channel Blocker, NNRTI—Non-Nucleoside Reverse Transcriptase Inhibitor.

## Data Availability

The original contributions presented in this study are included in the article. Further inquiries can be directed to the corresponding author.

## Abbreviations

The following abbreviations are used in this manuscript:

ACR	American College of Rheumatology
ACTH	Adrenocorticotropic hormone
ADA	Adenosine deaminase
AICAR	5-aminoimidazole-4-carboxamide ribonucleotide
APTT	activated partial thromboplastin time
ATIC	AICAR transformylase
ATLOs	Arterial tertiary lymphoid organs
BAFF	B-cell activating factor
bDMARDs	biologic disease-modifying antirheumatic drugs
BSR	British Society for Rheumatology
CRP	C-reactive protein
csDMARDs	Conventional synthetic disease-modifying antirheumatic drugs
CTX	C-terminal telopeptide of type I collagen
CYP3A4	Cytochrome P450
DAMPs	Damage-associated molecular patterns
DCs	Dendritic cells
DHFR	Dihydrofolate reductase
DHODH	Dihydroorotate dehydrogenase
DOAJ	Directory of open access journals
eGFR	Glomerular filtration rate
ESR	Erythrocyte sedimentation rate
ET-1	Endothelin-1
EULAR	European Alliance of Associations for Rheumatology
FPGS	Folylpolyglutamate synthetase
GC/GCs	Glucocorticoids
GCA	Giant cell arteritis
HCQ	Hydroxychloroquine
HEVs	High endothelial venules
HPA	Hypothalamic–pituitary–adrenal
HRQoL	Health-related quality of life
HSD-1	11β-hydroxysteroid dehydrogenase type 1
ICAM-1	Adhesion molecules
IFN-γ	Interferon-gamma
IL-1β	Interleukin-1β
IL-6	Interleukin-6
IL-8	Interleukin-8
IL-12	Interleukin-12
IL-17	Interleukin-17
IMPDH	Inosine monophosphate dehydrogenase
iNOS	Inducible nitric oxide synthase
JAK-STAT	Janus kinase-signal transducer and activator of transcription
JAKi	Janus kinase inhibitors
JNK	c-Jun N-terminal kinase
LF	Leflunomide
MACE	Major adverse cardiovascular events
MMF	Mycophenolate mofetil
MMPs	Matrix metalloproteinases
MPA	Mycophenolic acid
MTX	Methotrexate
MTX-PGs	Methotrexate polyglutamates
NETs	Neutrophil extracellular traps
PAMPs	Pathogen-associated molecular patterns
PD	Pharmacodynamic
PD-L1	Programmed death-ligand 1
PDGF	Platelet-derived growth factor
PET	Positron Emission Tomography
PINP	Procollagen type I N-terminal propeptide
PK	Pharmacokinetic
PMR	Polymyalgia rheumatica
PMR-AS	PMR Activity Scale
RFC1	Reduced folate carrier 1
ROS	Reactive oxygen species
RSV	Respiratory syncytial virus
SAM	S-adenosylmethionine
SASP	Senescence-associated secretory phenotype
STAT	Signal transducers and activators of transcription
TLA	Three letter acronym
TLRs	Toll-like receptors
TNF-α	Tumor necrosis factor-alpha
Tregs	Regulatory T cells
tsDMARDs	Targeted therapies based on small-molecule drugs
VCAM-1	Adhesion molecules
VEGF	Vascular endothelial growth factor
VSMCs	Vascular smooth muscle cells
VZV	Varicella-Zoster Virus

## References

[1] Castelo-Branco C., Soveral I. (2014). The Immune System and Aging: A Review. Gynecol. Endocrinol..

[2] Fulop T., Larbi A., Pawelec G., Khalil A., Cohen A.A., Hirokawa K., Witkowski J.M., Franceschi C. (2023). Immunology of Aging: The Birth of Inflammaging. Clin. Rev. Allergy Immunol..

[3] Schmeer C., Kretz A., Wengerodt D., Stojiljkovic M., Witte O.W. (2019). Dissecting Aging and Senescence—Current Concepts and Open Lessons. Cells.

[4] Bektas A., Schurman S.H., Sen R., Ferrucci L. (2017). Human T Cell Immunosenescence and Inflammation in Aging. J. Leukoc. Biol..

[5] Liu Z., Liang Q., Ren Y., Guo C., Ge X., Wang L., Cheng Q., Luo P., Zhang Y., Han X. (2023). Immunosenescence: Molecular Mechanisms and Diseases. Signal Transduct. Target. Ther..

[6] Youssef J., Novosad S.A., Winthrop K.L. (2016). Infection Risk and Safety of Corticosteroid Use. Rheum. Dis. Clin. N. Am..

[7] Xu M., Tchkonia T., Ding H., Ogrodnik M., Lubbers E.R., Pirtskhalava T., White T.A., Johnson K.O., Stout M.B., Mezera V. (2015). JAK Inhibition Alleviates the Cellular Senescence-Associated Secretory Phenotype and Frailty in Old Age. Proc. Natl. Acad. Sci. USA.

[8] Paroli M., Caccavale R., Accapezzato D. (2024). Giant Cell Arteritis: Advances in Understanding Pathogenesis and Implications for Clinical Practice. Cells.

[9] Henderson D., Eslamian G., Poon D., Crabb S., Jones R., Sankey P., Kularatne B., Linch M., Josephs D. (2020). Immune Checkpoint Inhibitor Induced Large Vessel Vasculitis. BMJ Case Rep..

[10] Deng J., Younge B.R., Olshen R.A., Goronzy J.J., Weyand C.M. (2010). Th17 and Th1 T-Cell Responses in Giant Cell Arteritis. Circulation.

[11] Ciccia F., Rizzo A., Ferrante A., Guggino G., Croci S., Cavazza A., Salvarani C., Triolo G. (2017). New Insights into the Pathogenesis of Giant Cell Arteritis. Autoimmun. Rev..

[12] Lyons H.S., Quick V., Sinclair A.J., Nagaraju S., Mollan S.P. (2020). A New Era for Giant Cell Arteritis. Eye.

[13] Esen I., Jiemy W.F., Van Sleen Y., Van Der Geest K.S.M., Sandovici M., Heeringa P., Boots A.M.H., Brouwer E. (2021). Functionally Heterogenous Macrophage Subsets in the Pathogenesis of Giant Cell Arteritis: Novel Targets for Dis-ease Monitoring and Treatment. J. Clin. Med..

[14] Planas-Rigol E., Terrades-Garcia N., Corbera-Bellalta M., Lozano E., Alba M.A., Segarra M., Espígol-Frigolé G., Prieto-González S., Hernández-Rodríguez J., Preciado S. (2017). Endothelin-1 Promotes Vascular Smooth Muscle Cell Migration across the Artery Wall: A Mechanism Contributing to Vascular Remodelling and Intimal Hyperplasia in Giant-Cell Arteritis. Ann. Rheum. Dis..

[15] Lozano E., Segarra M., García-Martínez A., Hernández-Rodríguez J., Cid M.C. (2008). Imatinib Mesylate Inhibits in Vitro and Ex Vivo Biological Responses Related to Vascular Occlusion in Giant Cell Arteritis. Ann. Rheum. Dis..

[16] Kaiser M., Weyand C.M., Björnsson J., Goronzy J.J. (1998). Platelet-Derived Growth Factor, Intimal Hyperplasia, and Ischemic Complications in Giant Cell Arteritis. Arthritis Rheum..

[17] Guggino G., Ferrante A., Macaluso F., Triolo G., Ciccia F. (2018). Pathogenesis of Polymyalgia Rheumatica. Reumatismo.

[18] Meliconi R., Pulsatelli L., Uguccioni M., Salvarani C., Macchioni P., Melchiorri C., Focherini M.C., Frizziero L., Facchini A. (1996). Leukocyte Infiltration in Synovial Tissue from the Shoulder of Patients with Polymyalgia Rheumatica. Arthritis Rheum. Off. J. Am. Coll. Rheumatol..

[19] Álvarez Rodríguez L., López-Hoyos M., Mata C., Fontalba A., Calvo Alen J., Marín M.J., Fernández-Luna J.L., Aguero Balbín J., Aranzamendi Zaldunbide M., Blanco R. (2011). Expression and Function of Toll-like Receptors in Peripheral Blood Mononuclear Cells of Patients with Polymyalgia Rheumatica and Giant Cell Arteritis. Ann. Rheum. Dis..

[20] Meliconi R., Pulsatelli L., Dolzani P., Boiardi L., Macchioni P., Salvarani C., Silvestri T., Frizziero L., Facchini A. (2000). Vascular Endothelial Growth Factor Production in Polymyalgia Rheumatica. Arthritis Rheum..

[21] Dejaco C., Duftner C., Al-Massad J., Wagner A.D., Park J.-K., Fessler J., Aigelsreiter A., Hafner F., Vega S., Sterlacci W. (2013). NKG2D Stimulated T-Cell Autoreactivity in Giant Cell Arteritis and Polymyalgia Rheumatica. Ann. Rheum. Dis..

[22] Samson M., Audia S., Fraszczak J., Trad M., Ornetti P., Lakomy D., Ciudad M., Leguy V., Berthier S., Vinit J. (2012). Th1 and Th17 Lymphocytes Expressing CD161 Are Implicated in Giant Cell Arteritis and Polymyalgia Rheumati-ca Pathogenesis. Arthritis Rheum..

[23] van der Geest K.S.M., Abdulahad W.H., Chalan P., Rutgers A., Horst G., Huitema M.G., Roffel M.P., Roozendaal C., Kluin P.M., Bos N.A. (2014). Disturbed B Cell Homeostasis in Newly Diagnosed Giant Cell Arteritis and Poly-myalgia Rheumatica. Arthritis Rheumatol..

[24] Eriksson P., Skoglund O., Hemgren C., Sjöwall C. (2023). Clinical Experience and Safety of Janus Kinase Inhibitors in Giant Cell Arteritis: A Retrospective Case Series from Sweden. Front. Immunol..

[25] Choy E.H., Unizony S.H., Wells A.F., Dasgupta B., Buttgereit F., Tanaka Y. (2026). Understanding the Immunopatho-physiology of Polymyalgia Rheumatica: Implications for Treatment. Ann. Rheum. Dis..

[26] Zhang L., Li J., Yin H., Chen D., Li Y., Gu L., Fu Y., Chen J., Chen Z., Yang S. (2023). Efficacy and Safety of Tofacitinib in Patients with Polymyalgia Rheumatica: A Phase 2 Study. Ann. Rheum. Dis..

[27] Padjen I., Crnogaj M.R., Anić B. (2020). Conventional Disease-Modifying Agents in Rheumatoid Arthritis—A Review of Their Current Use and Role in Treatment Algorithms. Reumatologia.

[28] Breedveld F.C., Dayer J.M. (2000). Leflunomide: Mode of Action in the Treatment of Rheumatoid Arthritis. Ann. Rheum. Dis..

[29] Smolen J.S., Landewé R.B.M., Bergstra S.A., Kerschbaumer A., Sepriano A., Aletaha D., Caporali R., Edwards C.J., Hyrich K.L., Pope J.E. (2023). EULAR Recommendations for the Management of Rheumatoid Arthritis with Synthet-ic and Biological Disease-Modifying Antirheumatic Drugs: 2022 Update. Ann. Rheum. Dis..

[30] Al-Abdulkarim H., Sharma Y., Attar S.M., Husain W., Al-Homood I., Al Omari B., Mohamed O., Alsaqa’aby M., Jaheen A.M., Anwar A. (2024). Cost-Effectiveness Analysis of Upadacitinib as a Treatment Option for Patients with Rheumatoid Arthritis in the Kingdom of Saudi Arabia. J. Med. Econ..

[31] O’Shea J.J., Schwartz D.M., Villarino A.V., Gadina M., McInnes I.B., Laurence A. (2015). The JAK-STAT Pathway: Impact on Human Disease and Therapeutic Intervention. Annu. Rev. Med..

[32] Hammarén H.M., Virtanen A.T., Raivola J., Silvennoinen O. (2019). The Regulation of JAKs in Cytokine Signaling and Its Breakdown in Disease. Cytokine.

[33] Radtke S., Haan S., Jörissen A., Hermanns H.M., Diefenbach S., Smyczek T., Schmitz-Vandeleur H., Heinrich P.C., Behrmann I., Haan C. (2005). The Jak1 SH2 Domain Does Not Fulfill a Classical SH2 Function in Jak/STAT Signaling but Plays a Structural Role for Receptor Interaction and up-Regulation of Receptor Surface Expression. J. Biol. Chem..

[34] Saharinen P., Takaluoma K., Silvennoinen O. (2000). Regulation of the Jak2 Tyrosine Kinase by Its Pseudokinase Domain. Mol. Cell. Biol..

[35] Valentino L., Pierre J. (2006). JAK/STAT Signal Transduction: Regulators and Implication in Hematological Malignancies. Biochem. Pharmacol..

[36] Levy D.E., Darnell J.E. (2002). Stats: Transcriptional Control and Biological Impact. Nat. Rev. Mol. Cell Biol..

[37] Favoino E., Prete M., Catacchio G., Ruscitti P., Navarini L., Giacomelli R., Perosa F. (2021). Working and Safety Profiles of JAK/STAT Signaling Inhibitors. Are These Small Molecules Also Smart?. Autoimmun. Rev..

[38] Jamilloux Y., El Jammal T., Vuitton L., Gerfaud-Valentin M., Kerever S., Sève P. (2019). JAK Inhibitors for the Treatment of Autoimmune and Inflammatory Diseases. Autoimmun. Rev..

[39] Roskoski R. (2023). Deucravacitinib Is an Allosteric TYK2 Protein Kinase Inhibitor FDA-Approved for the Treatment of Psoriasis. Pharmacol. Res..

[40] Rathore U., Thakare D.R., Patro P., Agarwal V., Sharma A., Misra D.P. (2022). A Systematic Review of Clinical and Preclinical Evidences for Janus Kinase Inhibitors in Large Vessel Vasculitis. Clin. Rheumatol..

[41] Traves P.G., Murray B., Campigotto F., Galien R., Meng A., Di Paolo J.A. (2021). JAK Selectivity and the Implications for Clinical Inhibition of Pharmacodynamic Cytokine Signalling by Filgotinib, Upadacitinib, Tofacitinib and Baricitinib. Ann. Rheum. Dis..

[42] Cafaro G., Bartoloni E., Alunno A., Bistoni O., Cipriani S., Topini F., Gerli R. (2019). A Platelet’s Guide to Synovitis. Isr. Med. Assoc. J. IMAJ.

[43] Conrad K., Shoenfeld Y., Fritzler M.J. (2020). Precision Health: A Pragmatic Approach to Understanding and Addressing Key Factors in Autoimmune Diseases. Autoimmun. Rev..

[44] Nash P., Kerschbaumer A., Konzett V., Aletaha D., Dörner T., Fleischmann R., McInnes I., Primdahl J., Sattar N., Tanaka Y. (2025). Expert Consensus Statement on the Treatment of Immune-Mediated Inflammatory Diseases with Janus Kinase Inhibitors: 2024 Update. Ann. Rheum. Dis..

[45] Fox R.I., Herrmann M.L., Frangou C.G., Wahl G.M., Morris R.E., Strand V., Kirschbaum B.J. (1999). Mechanism of Action for Leflunomide in Rheumatoid Arthritis. Clin. Immunol..

[46] Xu X., Williams J.W., Gong H., Finnegan A., Chong A.S. (1996). Two Activities of the Immunosuppressive Metabolite of Leflunomide, A77 1726. Inhibition of Pyrimidine Nucleotide Synthesis and Protein Tyrosine Phosphorylation. Biochem. Pharmacol..

[47] Elder R.T., Xu X., Williams J.W., Gong H., Finnegan A., Chong A.S. (1997). The Immunosuppressive Metabolite of Leflunomide, A77 1726, Affects Murine T Cells through Two Biochemical Mechanisms. J. Immunol..

[48] Brown P.M., Pratt A.G., Isaacs J.D. (2016). Mechanism of Action of Methotrexate in Rheumatoid Arthritis, and the Search for Biomarkers. Nat. Rev. Rheumatol..

[49] Cronstein B.N., Naime D., Ostad E. (1993). The Antiinflammatory Mechanism of Methotrexate. Increased Adenosine Release at Inflamed Sites Diminishes Leukocyte Accumulation in an in Vivo Model of Inflammation. J. Clin. Investig..

[50] Montesinos M.C., Desai A., Delano D., Chen J., Fink J.S., Jacobson M.A., Cronstein B.N. (2003). Adenosine A2A or A3 Receptors Are Required for Inhibition of Inflammation by Methotrexate and Its Analog MX-68. Arthritis Rheum..

[51] Genestier L., Paillot R., Fournel S., Ferraro C., Miossec P., Revillard J.P. (1998). Immunosuppressive Properties of Methotrexate: Apoptosis and Clonal Deletion of Activated Peripheral T Cells. J. Clin. Investig..

[52] Spurlock C.F., Tossberg J.T., Fuchs H.A., Olsen N.J., Aune T.M. (2012). Methotrexate Increases Expression of Cell Cycle Checkpoint Genes via JNK Activation. Arthritis Rheum..

[53] Allison A.C., Eugui E.M. (2000). Mycophenolate Mofetil and Its Mechanisms of Action. Immunopharmacology.

[54] Quéméneur L., Flacher M., Gerland L.-M., Ffrench M., Revillard J.-P., Bonnefoy-Berard N. (2002). Mycophenolic Acid Inhibits IL-2-Dependent T Cell Proliferation, but Not IL-2-Dependent Survival and Sensitization to Apoptosis. J. Immunol..

[55] Shimizu H., Takahashi M., Takeda S.-I., Inoue S., Fujishiro J., Hakamata Y., Kaneko T., Murakami T., Takeuchi K., Takeyoshi I. (2004). Mycophenolate Mofetil Prevents Transplant Arteriosclerosis by Direct Inhibition of Vascular Smooth Muscle Cell Proliferation. Transplantation.

[56] Hatakeyama K., Harada T., Kagamiyama H. (1992). IMP Dehydrogenase Inhibitors Reduce Intracellular Tetrahydrobiop-terin Levels through Reduction of Intracellular GTP Levels. Indications of the Regulation of GTP Cyclohydrolase I Activity by Restriction of GTP Availability in the Cells. J. Biol. Chem..

[57] Loricera J., Tofade T., Prieto-Peña D., Romero-Yuste S., De Miguel E., Riveros-Frutos A., Ferraz-Amaro I., Labrador E., Maiz O., Becerra E. (2024). Effectiveness of Janus Kinase Inhibitors in Relapsing Giant Cell Arteritis in Real-World Clinical Practice and Review of the Literature. Arthritis Res. Ther..

[58] Blockmans D., Penn S.K., Setty A.R., Schmidt W.A., Rubbert-Roth A., Hauge E.M., Keen H.I., Ishii T., Khalidi N., Dejaco C. (2025). A Phase 3 Trial of Upadacitinib for Giant-Cell Arteritis. N. Engl. J. Med..

[59] Chay T., Patel T., Dumaru N., Maddukuri S., Haddad R.R., Battula N.S., Mohammed L. (2025). A Systematic Review of the Use of Janus Kinase Inhibitors in Large Vessel Vasculitis. Cureus.

[60] Ishihara R., Watanabe R., Shiomi M., Fujita Y., Katsushima M., Fukumoto K., Yamada S., Hashimoto M. (2025). The Type I Interferon Axis in Systemic Autoimmune Diseases: From Molecular Pathways to Targeted Therapy. Biomolecules.

[61] Mahr A.D., Jover J.A., Spiera R.F., Hernández-García C., Fernández-Gutiérrez B., LaValley M.P., Merkel P.A. (2007). Adjunctive Methotrexate for Treatment of Giant Cell Arteritis: An Individual Patient Data Meta-analysis. Arthritis Rheum..

[62] Castañeda S., Prieto-Peña D., Vicente-Rabaneda E.F., Triguero-Martínez A., Roy-Vallejo E., Atienza-Mateo B., Blanco R., González-Gay M.A. (2022). Advances in the Treatment of Giant Cell Arteritis. J. Clin. Med..

[63] De Silva M., Hazleman B.L. (1986). Azathioprine in Giant Cell Arteritis/Polymyalgia Rheumatica: A Double-Blind Study. Ann. Rheum. Dis..

[64] Diamantopoulos A.P., Hetland H., Myklebust G. (2013). Leflunomide as a Corticosteroid-Sparing Agent in Giant Cell Arteritis and Polymyalgia Rheumatica: A Case Series. BioMed Res. Int..

[65] Karabayas M., Dospinescu P., Fluck N., Kidder D., Fordyce G., Hollick R.J., De Bari C., Basu N. (2020). Evaluation of Adjunctive Mycophenolate for Large Vessel Giant Cell Arteritis. Rheumatol. Adv. Pract..

[66] De Boysson H., Boutemy J., Creveuil C., Ollivier Y., Letellier P., Pagnoux C., Bienvenu B. (2013). Is There a Place for Cyclophosphamide in the Treatment of Giant-Cell Arteritis? A Case Series and Systematic Review. Semin. Arthritis Rheum..

[67] Ma X., Yang F., Wu J., Xu B., Jiang M., Sun Y., Sun C., Yu Y., Xu D., Xiao L. (2023). Efficacy and Safety of Tofacitinib in Patients with Polymyalgia Rheumatica (EAST PMR): An Open-Label Randomized Controlled Trial. PLoS Med..

[68] Gu J., Yang M., Zhang B., Wang H. (2023). Efficacy of JAK Inhibitors versus DMARDs in the Treatment of Polymyalgia Rheumatica in China. Int. J. Gen. Med..

[69] Saraux A., Carvajal Alegria G., Dernis E., Roux C., Richez C., Tison A., Quere B., Jousse-Joulin S., Guellec D., Marhadour T. (2025). Baricitinib in Early Polymyalgia Rheumatica (BACHELOR): A Randomised, Double-Blind, Pla-cebo-Controlled, Parallel-Group Trial. Lancet Rheumatol..

[70] García-Porrúa C., Heras-Recuero E., Blázquez-Sánchez T., Torres-Roselló A., Castañeda S., González-Gay M.Á. (2024). Traditional and Emerging Strategies for Managing Polymyalgia Rheumatica: Insights into New Treatments. J. Clin. Med..

[71] Lundberg I.E., Sharma A., Turesson C., Mohammad A.J. (2022). An Update on Polymyalgia Rheumatica. J. Intern. Med..

[72] Colombo M.G., Wetzel A.-J., Haumann H., Dally S., Kirtschig G., Joos S. (2022). Polymyalgia Rheumatica. Dtsch. Ärztebl. Int..

[73] Caporali R., Cimmino M.A., Ferraccioli G., Gerli R., Klersy C., Salvarani C., Montecucco C., for the Systemic Vasculitis Study Group of the Italian Society for Rheumatology (2004). Prednisone plus Methotrexate for Polymyalgia Rheumatica: A Randomized, Double-Blind, Placebo-Controlled Trial. Ann. Intern. Med..

[74] Ferraccioli G., Salaffi F., De Vita S., Casatta L., Bartoli E. (1996). Methotrexate in Polymyalgia Rheumatica: Preliminary Results of an Open, Randomized Study. J. Rheumatol..

[75] Van Der Veen M.J., Dinant H.J., Van Booma-Frankfort C., Van Albada-Kuipers G.A., Bijlsma J.W. (1996). Can Methotrex-ate Be Used as a Steroid Sparing Agent in the Treatment of Polymyalgia Rheumatica and Giant Cell Arteritis?. Ann. Rheum. Dis..

[76] Adizie T., Christidis D., Dharmapaliah C., Borg F., Dasgupta B. (2012). Efficacy and Tolerability of Leflunomide in Difficult-to-Treat Polymyalgia Rheumatica and Giant Cell Arteritis: A Case Series: Efficacy and Tolerability of Leflunomide. Int. J. Clin. Pract..

[77] Vinicki J.P., Cusa A., Domingo D., Velasco Zamora J.L., Magri S., Brigante A., Schmid M.M., Ávila P., Zamora N., Sorrentino L. (2024). Effectiveness of Methotrexate and Leflunomide as Corticoid-Sparing Drugs in Patients with Polymyalgia Rheumatica. Rheumatol. Adv. Pract..

[78] Bellaire S., Walzer M., Wang T., Krauwinkel W., Yuan N., Marek G.J. (2019). Safety, Pharmacokinetics, and Pharmaco-dynamics of ASP 3662, a Novel 11β-Hydroxysteroid Dehydrogenase Type 1 Inhibitor, in Healthy Young and Elderly Subjects. Clin. Transl. Sci..

[79] Buttgereit F., Everding A., Andreica I., Kellner H.L., Schuch F., Weyand C., Stewart P.M., Merkel P.A., Dejaco C., Czerwiec F.S. (2025). Effects of Clofutriben, a Selective 11β-Hydroxysteroid Dehydrogenase Type 1 Inhibitor, on the Efficacy and Toxicity of Prednisolone in Patients with Polymyalgia Rheumatica: A Single-Blind Controlled Trial with Sequential Cohorts. Ann. Rheum. Dis..

[80] Othonos N., Pofi R., Arvaniti A., White S., Bonaventura I., Nikolaou N., Moolla A., Marjot T., Stimson R.H., Van Beek A.P. (2023). 11β-HSD1 Inhibition in Men Mitigates Prednisolone-Induced Adverse Effects in a Proof-of-Concept Randomised Double-Blind Placebo-Controlled Trial. Nat. Commun..

[81] Fenton C.G., Doig C.L., Fareed S., Naylor A., Morrell A.P., Addison O., Wehmeyer C., Buckley C.D., Cooper M.S., Lavery G.G. (2019). 11β-HSD1 Plays a Critical Role in Trabecular Bone Loss Associated with Systemic Glucocorticoid Therapy. Arthritis Res. Ther..

[82] Teng L., Li L., Cui D., An R., Jin J. (2024). Polymyalgia Rheumatica and Giant Cell Arteritis: A Bidirectional Mendelian Randomization Study. Medicine.

[83] Raheel S., Shbeeb I., Crowson C.S., Matteson E.L. (2017). Epidemiology of Polymyalgia Rheumatica 2000–2014 and Examination of Incidence and Survival Trends Over 45 Years: A Population-Based Study. Arthritis Care Res..

[84] Muller S., Hider S.L., Helliwell T., Lawton S., Barraclough K., Dasgupta B., Zwierska I., Mallen C.D. (2016). Characteris-ing Those with Incident Polymyalgia Rheumatica in Primary Care: Results from the PMR Cohort Study. Arthritis Res. Ther..

[85] Kawka L., Chevet B., Arnaud L., Becker G., Carvajal Alegria G., Felten R. (2024). The Pipeline of Immunomodulatory Therapies in Polymyalgia Rheumatica and Giant Cell Arteritis: A Systematic Review of Clinical Trials. Autoimmun. Rev..

[86] Charlton R. (2012). Optimal Management of Giant Cell Arteritis and Polymyalgia Rheumatica. Ther. Clin. Risk Manag..

[87] Schmidt J., Warrington K.J. (2011). Polymyalgia Rheumatica and Giant Cell Arteritis in Older Patients: Diagnosis and Pharmacological Management. Drugs Aging.

[88] Bauters T., Dadkhah A., Sureda A., Cor-bacioglu S., Greco R., Kröger N., Carreras E. (2024). Clinically Relevant Drug Interactions in HCT. The EBMT Handbook.

[89] Shawky A.M., Almalki F.A., Abdalla A.N., Abdelazeem A.H., Gouda A.M. (2022). A Comprehensive Overview of Globally Approved JAK Inhibitors. Pharmaceutics.

[90] Dejaco C., Singh Y.P., Perel P., Hutchings A., Camellino D., Mackie S., Abril A., Bachta A., Balint P., Barraclough K. (2015). 2015 Recommendations for the Management of Polymyalgia Rheumatica: A European League Against Rheumatism/American College of Rheumatology Collaborative Initiative. Arthritis Rheumatol..

[91] Joensuu J.T., Aaltonen K.J., Aronen P., Sokka T., Puolakka K., Tuompo R., Korpela M., Vasala M., Ilva K., Nordström D. (2016). Cost-Effectiveness of Biologic Compared with Conventional Synthetic Disease-Modifying An-ti-Rheumatic Drugs in Patients with Rheumatoid Arthritis: A Register Study. Rheumatology.

[92] Mikaeili B., Alqahtani Z.A., Hincapie A.L., Guo J.J. (2025). Safety of Janus Kinase Inhibitors in Rheumatoid Arthritis: A Disproportionality Analysis Using FAERS Database from 2012 to 2022. Clin. Rheumatol..

[93] Song G.G., Lee Y.H. (2021). Methotrexate for Treating Polymyalgia Rheumatica: A Meta-Analysis of Randomized Controlled Trials. Int. J. Clin. Pharmacol. Ther..

[94] Morrison T., Foster E., Dougherty J., Barton J. (2022). Shared Decision Making in Rheumatology: A Scoping Review. Semin. Arthritis Rheum..

[95] Yap A.F., Thirumoorthy T., Kwan Y.H. (2016). Systematic Review of the Barriers Affecting Medication Adherence in Older Adults. Geriatr. Gerontol. Int..

[96] Clegg A., Young J., Iliffe S., Rikkert M.O., Rockwood K. (2013). Frailty in Elderly People. The Lancet.

[97] Rockwood K. (2005). A Global Clinical Measure of Fitness and Frailty in Elderly People. Can. Med. Assoc. J..

[98] Ytterberg S.R., Bhatt D.L., Mikuls T.R., Koch G.G., Fleischmann R., Rivas J.L., Germino R., Menon S., Sun Y., Wang C. (2022). Cardiovascular and Cancer Risk with Tofacitinib in Rheumatoid Arthritis. N. Engl. J. Med..

[99] Bechman K., Song K., Abhishek A., Adas M., Ahmed A., Bray L., Davidson A., Deepak S., Dey M., De Vere H. (2026). The 2025 British Society for Rheumatology Guideline for the Prescription and Monitoring of Conventional Synthetic Disease-Modifying Anti-Rheumatic Drugs. Rheumatology.

[100] Ibrahim K., Cox N.J., Stevenson J.M., Lim S., Fraser S.D.S., Roberts H.C. (2021). A Systematic Review of the Evidence for Deprescribing Interventions among Older People Living with Frailty. BMC Geriatr..

